# Supplementary material for: Intranasally Administered Exosomes from Umbilical Cord Stem Cells Have Preventive Neuroprotective Effects and Contribute to Functional Recovery after Perinatal Brain Injury
Source: Cells. 2019 Aug 8;8(8):855. doi: 10.3390/cells8080855 (PMC6721675; doi:10.3390/cells8080855)
Supplement: Supplementary file 1 [file cells-08-00855-s001.pdf]

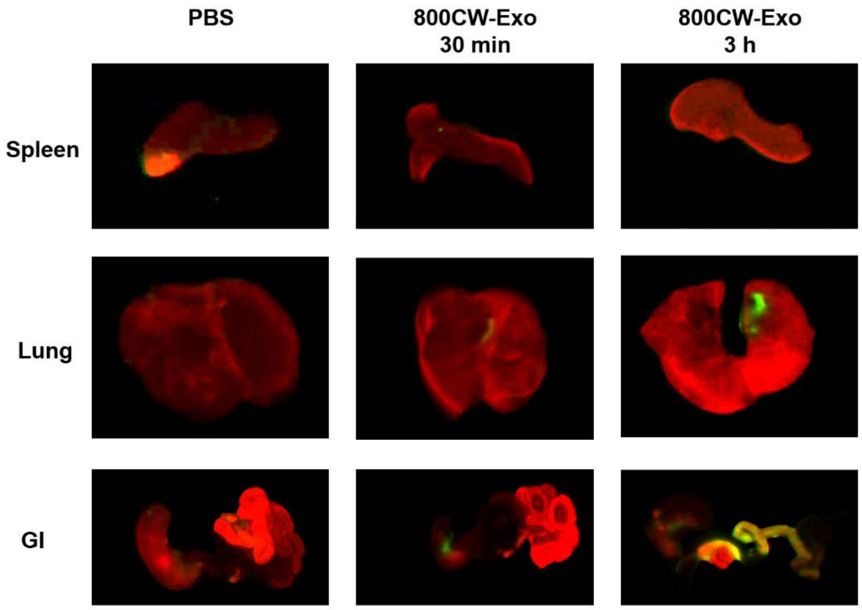


**Figure S1.** Ectopic accumulation of exosomes after intranasal administration of mesenchymal stromal cell-derived exosomes. Representative images of the spleen, the lung and the gastro-intestinal tract (GI) from rats with PBI 30 minutes and 3 hours after intranasal administration of either PBS or IRDye® 800CW-labeled exosomes (green) in PBS. Background tissue autofluorescence is depicted in red.
